# Network Pharmacology Identifies Therapeutic Targets and the Mechanisms of Glutathione Action in Ferroptosis Occurring in Oral Cancer

**DOI:** 10.3389/fphar.2022.851540

**Published:** 2022-03-14

**Authors:** Chen Huang, Lei Zhan

**Affiliations:** ^1^ The Center for Data Science in Health and Medicine, Business School, Qingdao University, Qingdao, China; ^2^ Department of Ophthalmology, The Second People’s Hospital of Guilin, Guilin, China

**Keywords:** oral cancer, GSH, ferroptosis, network pharmacology, targets

## Abstract

Oral cancer (OC) is one of the most pernicious cancers with increasing incidence and mortality worldwide. Surgery is the primary approach for the treatment of early-stage OC, which reduces the quality of life of the patients. Therefore, there is an urgent need to discover novel treatments for OC. Targeting ferroptosis to induce cell death through the modulation of lipid oxidation has been used as a new approach to treat many cancers. Glutathione (GSH) is a coenzyme factor of GSH peroxidase 4, and it carries potential applicability in treating OC. By using network pharmacology and molecular docking followed by systematic bioinformatic analysis, we aimed to study GSH-targeting ferroptosis to treat OC. We identified 14 core molecular targets, namely, EGFR, PTGS2, HIF1A, VEGFA, TFRC, SLC2A1, CAV1, CDKN2A, SLC3A2, IFNG, NOX4, DDIT4, CA9, and DUSP1, involved in ferroptosis that were targeted by GSH for OC treatment. Functional characterization of these molecular targets showed their importance in the control of cell apoptosis, cell proliferation, and immune responses through various kinase activities such as the mitogen-activated protein kinase activity (e.g., ERK1 and ERK2 cascades) and modulation of TOR signaling (e.g., the HIF-1 signaling pathway). Molecular docking further revealed the direct binding of GSH with EGFR, PTGS2, and HIF1A proteins. These findings provide a novel insight into the targets of GSH in ferroptosis as well as possible molecular mechanisms involved, suggesting the possible use of GSH as a combined therapy for treating OC.

## Introduction

Oral cancer (OC) is a type of malignant tumor that occurs in the oral cavity; it includes tongue cancer, gum cancer, palate cancer, oropharyngeal cancer, and lip cancer (Wong and Wiesenfeld 2018). OC is one of the most pernicious cancers reported in the world, accounting for around 2% of all new cases (Sung et al., 2021). In recent decades, the incidence and mortality of OC have shown markedly increasing trends (Chen et al., 2016). Potential risk factors for OC include smoking, drinking, poor oral hygiene, malnutrition, environmental impact, genetic factors, and infectious diseases (Shield et al., 2017). Tumor formation in OC results from abnormal activation patterns of proto- and anti-oncogenes and epigenetic modification (Osan et al., 2021). Early clinical detection of OC may be difficult to achieve because of insidious conditions and anatomical characteristics prior to initial treatment (Dhanuthai et al., 2018). Clinically, surgery is prioritized for early-stage OC with better treatment effects (D'Souza and Kumar 2020). However, chemotherapy is a palliative regimen, and its efficacy is not yet satisfactory for patients with advanced OC (Mohan et al., 2021). Therefore, markers for early detection of oral malignancies and alternative treatment agents are necessary. Ferroptosis, a type of programmed cell death dependent on iron and activated by lipid peroxidation, is closely related to cancer development (Nie et al., 2022). Initiation and induction of ferroptosis can cause abnormal function of mitochondria and peroxidation-based lipotoxicity, resulting in regulation of tumor formation (Wu et al., 2020). It is reported that malignant tumor cells contain high levels of iron elements for anarchic cell proliferation and tumor growth (Tang et al., 2021a). Therefore, targeting ferroptosis may be a promising approach for OC treatment. Glutathione (GSH) is a bioactive substance involved in cellular metabolism that physiologically functions to protect the body against lesions induced by reducing agents (Forman et al., 2009). GSH is a coenzyme factor of GSH peroxidase 4 (GPx4), an essential reaction substrate for the degradation of lipid peroxides. As a key enzyme regulating ferroptosis, GPx4 can inhibit the occurrence of ferroptosis by catalyzing the reduction of lipid peroxides (Xuan et al., 2021). Patients with OC have markedly low levels of superoxide dismutase, GSH peroxidase, and GSH transferase (Sushma et al., 2021). An *in vitro* study demonstrated that enhanced intracellular GSH activity induced by natural compounds might have anti-oral cancer action by modulating oxidative stress, autophagy, and cell death (Contant et al., 2021). Although the physiological function of GSH in OC and underlying molecular mechanisms are well-reported, pharmacological mechanisms of GSH against OC remain unclear, especially ferroptosis-associated signaling pathways. Recently, network pharmacology-based discovery of individual compounds that act against dysregulated disorders, including malignant cancer (Li et al., 2021a), has been demonstrated (Li et al., 2020). Using a network pharmacology screening approach, our previous study demonstrated the core targets and therapeutic mechanisms of the GSH action against the cleft lip (Li et al., 2021b). In this study, available bioinformatic data of GSH were processed and studied for the potential efficacy against OC *via* multi-step network pharmacology and molecular docking approach, revealing ferroptosis-associated biotargets and signaling mechanisms.

## Material and Methods

### Identification of Common Oral Cancer-, Ferroptosis-, and Glutathione-Associated Genes

The Cancer Genome Atlas (TCGA) database (https://portal.gdc.cancer.gov/) was used to determine OC-associated genes. Using the limma package of R in the Bioconductor software, genes with FDR <0.05 and |log2 fold change| > 1 were considered as OC-associated genes (Ritchie et al., 2015). The FerrDb database was then used to search for ferroptosis-associated genes (Zhou and Bao 2020). For GSH-associated genes, the chemical structure of GSH, C (CC(=O)NC(CS)C (=O)NCC(=O)O)C(C(=O)O)N, was obtained from the traditional Chinese medicine system pharmacology database (Ru et al., 2014) and used to determine the pharmacological targets of GSH using various online tools and databases including SwissTargetPrediction (Daina et al., 2019), SuperPred (Nickel et al., 2014), TargetNet (Yao et al., 2016), Batman (Liu et al., 2016), DrugBank (Wishart et al., 2018), and BindingDB (Liu et al., 2007). The target genes were subjected to UniProt for human database correction. Subsequently, all targets of OC, ferroptosis, and GSH were overlapped to obtain the common targets.

### Protein Network Involved in Glutathione Action Against Oral Cancer Through Ferroptosis

The common targets were subjected to a protein–protein interaction (PPI) network analysis by using the STRING (Version 11.0) database (Szklarczyk et al., 2019). The network analyzer in Cytoscape v3.7.2 was set under median or maximum degrees of freedom; the core targets were obtained under the upper limit of the screening range with a maximum degree value of the topology data, and the lower limit was twice the median degree of freedom (Shannon et al., 2003).

### Gene Ontology and Pathway Enrichment Analysis of CA028 Action Against Oral Cancer

The common targets were subjected to Bioconductor packages in the R-language software for Gene Ontology (GO) and Kyoto Encyclopedia of Genes and Genomes (KEGG) enrichment analysis. Then, the Cytoscape software was used to visualize the biological processes and signaling pathways involved in the GSH action against OC through ferroptosis (Shannon et al., 2003).

### Molecular Docking Analysis

The binding of GSH to its core targets including the epidermal growth factor receptor (EGFR), prostaglandin-endoperoxide synthase 2 (PTGS2), and hypoxia inducible factor 1 subunit alpha (HIF1A) was studied by molecular docking. The protein structures of EGFR, PTGS2, and HIF1A were obtained from the PDB database. Then, MGLTools 1.5.6 of AutoDock Vina and the ChemBio3D Draw tool were used to conduct the docking analysis (Morris et al., 2009; Trott and Olson 2010). The docking parameter setting was assessed according to the root mean square deviation (RMSD) of the ligand molecule. RMSD ≤ 4Å was the permissive threshold for the conformation of the ligand molecule.

## Results

### Identification of Glutathione Targets for Treating Oral Cancer Through Ferroptosis

**FIGURE 1:**
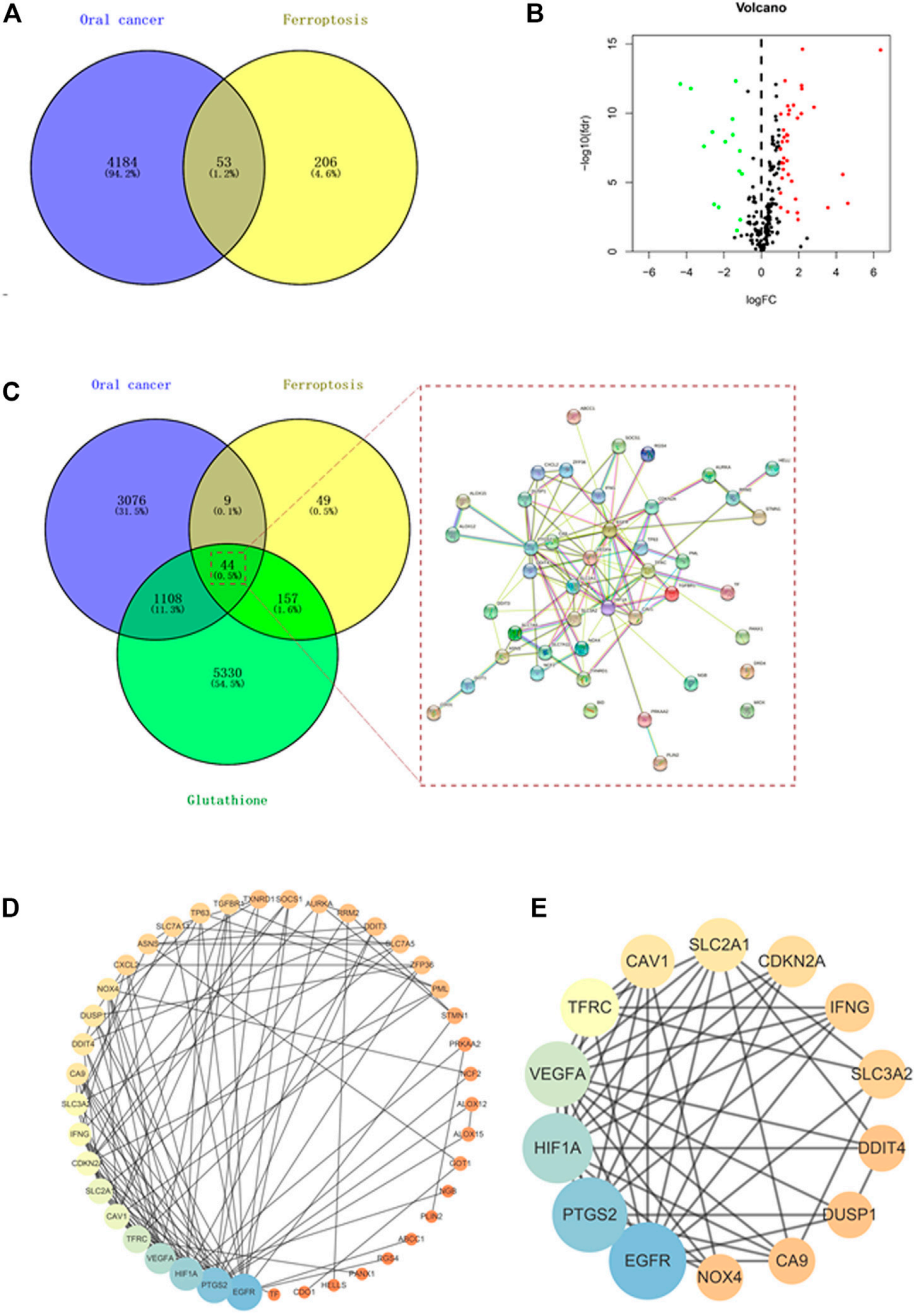
Network pharmacology identified the anti-oral cancer genes involved in targeting ferroptosis by glutathione (GSH). **(A)** Venn diagram showing the interacting genes associated with oral cancer (OC) and ferroptosis. **(B)** Volcano plot showing the differential expression of ferroptosis-associated genes in OC. The genes with FDR <0.05 and |log2 fold change| > 1 were considered as differentially expressed genes. Green dots represent downregulated genes, and red dots represent upregulated genes. **(C)** Venn diagram showing the interacting genes associated with OC, ferroptosis, and GSH. **(D)** Protein–protein interaction (PPI) of OC-, ferroptosis-, and GSH-associated genes. **(E)** Cytoscape analysis identified 14 core targets of GSH for treating OC by targeting ferroptosis.

By searching the FerrDb and GeneCards databases, 259 ferroptosis-associated targets were identified (Figure 1A). Additionally, using TCGA database, the comparison of gene sequences from 32 normal adults and 330 patients with OC identified 4,237 differentially expressed genes (DEGs) (Figure 1A). Among these, 53 DEGs including 38 upregulated genes and 15 downregulated genes overlapped with ferroptosis-associated targets (Figure 1B). Furthermore, 6639 GSH-associated target genes were identified by searching different databases, and 44 of them were found to be shared with OC- and ferroptosis-associated targets (Figure 1C). Then, the common targets were subjected to the STRING analysis to delineate the PPI involved in the GSH action against OC through ferroptosis (Figure 1D). By using the Cytoscape tool, 14 core targets, namely, PTGS2, EGFR, HIF1A, cyclin-dependent kinase inhibitor 2A (CDKN2A), vascular endothelial growth factor A (VEGFA), interferon gamma (IFNG), dual specificity phosphatase 1 (DUSP1), NADPH oxidase 4 (NOX4), solute carrier family 3 member 2 (SLC3A2), solute carrier family 2 member 1 (SLC2A1), caveolin 1 (CAV1), carbonic anhydrase 9 (CA9), transferrin receptor (TFRC), and DNA damage-inducible transcript 4 (DDIT4) were obtained. The median degree of freedom was 5.37, whereas the maximum degree of freedom was 19 (Figure 1E and Table 1).

**TABLE 1:** Information of GSH anti-oral cancer genes.

Gene name	Gene symbol	UniProt ID
Prostaglandin-endoperoxide synthase 2	PTGS2	P35354
Epidermal growth factor receptor	EGFR	P00533
Hypoxia inducible factor 1 subunit alpha	HIF1A	Q16665
Cyclin-dependent kinase inhibitor 2A	CDKN2A	P42771
Vascular endothelial growth factor A	VEGFA	P15692
Interferon gamma	IFNG	P01579
Dual specificity phosphatase 1	DUSP1	P28562
NADPH oxidase 4	NOX4	Q9NPH5
Solute carrier family 3 member 2	SLC3A2	P08195
Solute carrier family 2 member 1	SLC2A1	P11166
Caveolin 1	CAV1	Q03135
Carbonic anhydrase 9	CA9	Q16790
Transferrin receptor	TFRC	P02786
DNA damage inducible transcript 4	DDIT4	Q9NX09

### Glutathione Targeted Ferroptosis to Control Stress Responses and Cell Apoptosis of Oral Cancer

**FIGURE 2:**
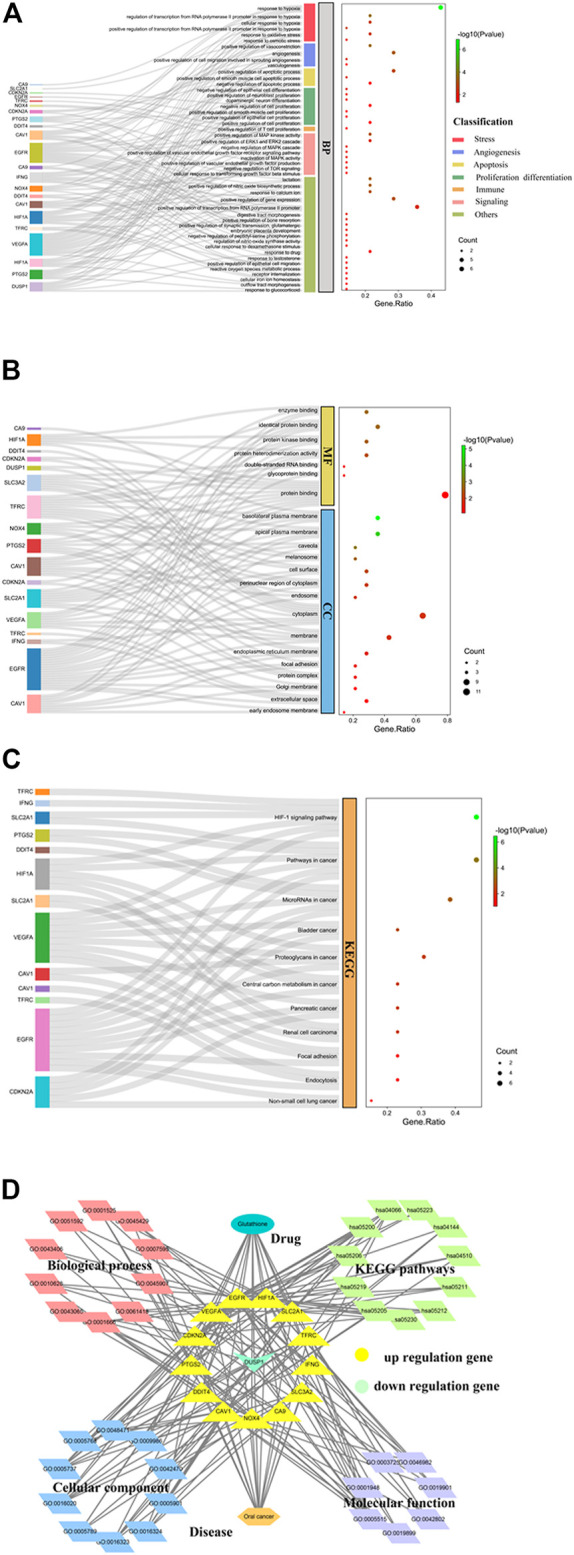
Functional characterization of glutathione (GSH)-targeting ferroptosis genes in oral cancer (OC). **(A)** Gene Ontology (GO) enrichment analysis highlighting the biological roles of GSH-targeting ferroptosis genes in stress responses, angiogenesis, apoptosis, cell proliferation, cell differentiation, immune responses, and cell signaling for treating OC. **(B)** GO enrichment analysis highlighting the molecular functions and cellular components of GSH-targeting ferroptosis genes for treating OC. **(C)** Kyoto Encyclopedia of Genes and Genomes (KEGG) pathway analysis showed the importance of target genes of GSH action in cell signaling pathways involved in OC. The size of the dots represents the number of genes, whereas the color of the dots represents the significance of the terms. **(D)** Complex network analysis using Cytoscape to visualize the biological processes and signaling pathways involved in the mechanism of GSH action against OC through ferroptosis.

GO enrichment analysis of the 14 core targets highlighted the biological processes related to stress responses such as oxidative stress and hypoxic stress (Figure 2A). Besides, altered angiogenesis through vasoconstriction and vasculogenesis was observed (Figure 2A). Furthermore, this could lead to the control of cell apoptosis, cell proliferation, and immune responses through many kinase activities and pathways, such as the mitogen-activated protein kinase (MAPK) activity, the extracellular signal-regulated kinase (ERK)1 and ERK2 cascades, and TOR signaling (Figure 2A). Molecular functional analysis of GO showed the involvement of the core targets in many binding activities such as enzyme binding, protein binding, protein kinase binding, glycoprotein binding, double-stranded RNA binding, and protein heterodimerization activity (Figure 2B). GO analysis also highlighted the contribution of core targets to different cellular components such as the endosome, cytoplasm membrane, Golgi membrane, and extracellular space (Figure 2B). KEGG pathway analysis revealed the control of cell signaling pathways such as the HIF-1 signaling pathway by GSH targets (Figure 2C). In addition, many cancer-related signaling pathways, including mRNAs in cancer, bladder cancer, proteoglycans in cancer, central carbon metabolism in cancer, pancreatic cancer, renal cell carcinoma, and non-small cell lung cancer, were highlighted in our analysis (Figure 2C). Taken together, our results show the importance of GSH against ferroptosis in OC through the regulation of many cancer-related biological processes and signaling pathways (Figure 2D).

### Direct Binding of Glutathione to Oral Cancer- and Ferroptosis-Associated Genes

**FIGURE 3:**
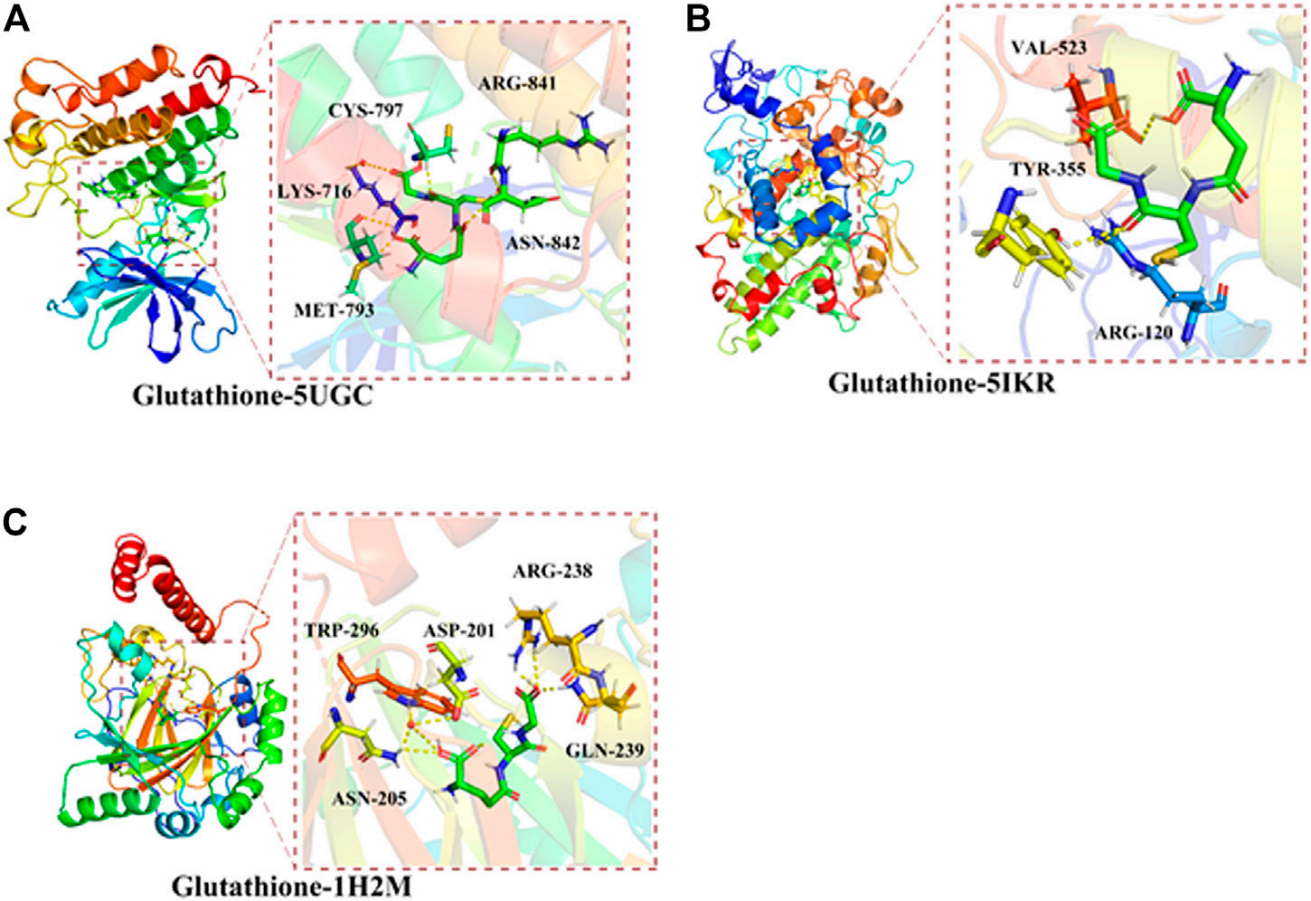
Direct binding of glutathione (GSH) to its targets against oral cancer through ferroptosis. Molecular docking analysis predicted the preferred orientation of hydrogen bonds between GSH and amino acid residues **(A)** CYS-797, MET-793, and LYS-716 of EGFR (PDB ID:5UGC) **(B)** TYR-355, ARG-120, and VAL-523 of PTGS2 (PDB ID: 5IKR) **(C)** ARG-238 and GLN-239 of HIF1A (PDB ID: 1H2M).

Molecular docking analysis was conducted to investigate the possible direct binding of GSH to its target proteins. The results showed that GSH had a high binding affinity to EGFR, PTGS2, and HIF1A (Figure 3). The protein structures of EGFR, PTGS2, and HIF1A were obtained from the PDB database. Furthermore, the AutoDock Vina program was used to determine the binding affinity between these proteins and GSH. The results demonstrated that GSH formed hydrogen bonds with amino acid residues CYS-797 (1.9 Å), MET-793 (2.5 Å), and LYS-716 (2.5 Å) of EGFR (PDB ID: 5UGC) of EGFR, and the free binding energy was −5.1 kcal/mol. For PTGS2 protein (PDB ID: 5IKR), GSH formed hydrogen bonds with amino acid residues TYR-355 (2.6 Å), ARG-120 (2.3 Å), and VAL-523 (2.4 Å) of PTGS2 (Figure 3B), and the free binding energy was -5.12 kcal/mol. Similar bonds were observed between GSH and amino acid residues ARG-238 (2.5 Å) and GLN-239 (1.8 Å) of HIF1A (PDB ID: 1H2M) (Figure 3C), with a free binding energy of −5.32 kcal/mol. Collectively, our data suggested direct binding of GSH to its target proteins EGFR, PTGS2, and HIF1A.

## Discussion

In the present study, we investigated the possible use of GSH in treating OC by targeting ferroptosis. Ferroptosis, a newly discovered type of cell death mediated by iron-dependent lipid peroxidation (Chen S. et al., 2021), is reported to be a promising approach in cancer therapy (Tang et al., 2021b). Oral squamous cell carcinoma was shown to have a ferroptosis-specific gene-expression signature, suggesting ferroptosis regulation may be clinically relevant for combination therapies (Gu et al., 2021). We hypothesized that GSH can be a potential drug for combination therapies of OC as it is a key player in cellular oxidation–reduction homeostasis and is closely associated with ferroptosis in various cancers (Elakkad et al., 2021; Liu et al., 2021; Xu et al., 2021).

By using network pharmacology, we identified 14 core targets of GSH action against OC by targeting ferroptosis. Bioinformatic analysis of these 14 targets suggested the role of GSH in cell apoptosis, cell proliferation, and immune responses through the regulation of MAPK, ERK, and TOR signaling pathways. MAPK was found to be involved in ferroptosis regulation in many human body functions (Nguyen et al., 2020). Activation of MAPK signaling induced ferroptosis in human pancreatic islet-cell clusters (Li and Leung 2020). In contrast, inhibition of MAPK signaling suppressed inflammation and oxidative stress of acute respiratory distress syndrome caused by ferroptosis (Wang et al., 2022). In other studies, MAPK signaling was found to control ferroptosis in various cancers including nasopharyngeal carcinoma, hepatocellular carcinoma, osteosarcoma, and non-small cell lung cancer through redox balance (Poursaitidis et al., 2017; Lv et al., 2020; Chang et al., 2021; He et al., 2021). More importantly, MAPK signaling was linked to many other cell signaling pathways mediating the carcinogenicity of OC. For instance, MAPK signaling regulated the viability of OC *via* the response gene FOS as a subunit of the AP-1 complex (Greer et al., 2022). Also, inhibition of the MAPK pathway diminished invasion and the migration ability in OC through the regulation of matrix metalloproteinase-2 activity (Pramanik and Mishra 2022). Besides the MAPK signaling, our results highlighted the involvement of ERK signaling in ferroptosis targeted by GSH. ERK signaling is associated with ferroptosis in many cancers, including multiple myeloma (Chen J. et al., 2021), endometrial carcinoma (Qin et al., 2021), hepatocellular carcinoma (Fei et al., 2021), and pancreatic cancer (Ye et al., 2020). Specifically, the ERK signaling pathway has been linked with ferroptosis and associated with the prognosis of OC (Chen R. et al., 2021). For TOR signaling, only limited studies have demonstrated its direct link to ferroptosis. A study by Ni et al. demonstrated that inhibition of mTOR overcame anticancer drug resistance by promoting ferroptosis in lung cancer cells (Ni et al., 2021). Another study on xenograft mouse models suggested that the combination of mTORC1 inhibition with ferroptosis induction resulted in tumor regression in PI3K-mutated breast cancer and PTEN-defective prostate cancer (Yi et al., 2020). Therefore, the use of GSH to target ferroptosis could be a promising combination therapy for treating OC.

In addition to network pharmacology, we applied molecular docking to investigate the possible direct binding of GSH to its target proteins involved in ferroptosis. Our results showed a strong binding affinity of GSH to EGFR, PTGS2, and HIFIA.

EGFR, a receptor tyrosine kinase, is a major regulator of cancer development (Ni et al., 2021). It coordinates with mTOR and MAPK signaling to regulate ferroptosis in cancer. For example, EGFR was associated with the mTOR pathway to mediate ferroptosis and apoptosis of ovarian cancer (Li et al., 2022). In addition, the EGFR tyrosine kinase inhibitor lapatinib regulated mTOR to promote ferroptosis in lung cancer cells (Ni et al., 2021). Another study of non-small cell lung cancer demonstrated that the blockade of EGFR or MAPK signaling protected the lung cancer cells from ferroptosis (Poursaitidis et al., 2017). Furthermore, it has been shown that ferroptosis can be targeted to treat EGFR-mutant lung cancer (Zhang et al., 2021). Additionally, driving EGFR could induce ferroptosis in hepatocellular carcinoma and glioblastoma (Kadioglu et al., 2021; Sun et al., 2021). PTGS2 is responsible for the prostanoid biosynthesis involved in inflammation and mitogenesis (Gómez-Valenzuela et al., 2021). It was reported that upregulation of PTGS2 induces the ferroptosis of colorectal cancer cells (Zhao and Chen 2021); thus, PTGS2 is considered as a marker of ferroptosis. The PTGS2 expression was associated with increased lipid peroxidation in gastric cancer cells (Guan et al., 2020). In another study, the PTGS2 expression modulated esophageal squamous cell carcinoma radiosensitivity by inhibiting ferroptosis (Feng et al., 2021). In addition, HIF1A was associated with increased tumor immunity and aggressive phenotypes in human cancers (Chen et al., 2020). Ferroptosis-related genes associated with the overall survival in patients with diffuse large B-cell lymphoma have also been reported (Chen H. et al., 2021).

In conclusion, we identified the targets of GSH in ferroptosis associated with OC, providing new mechanistic insights that may be clinically relevant for combination therapies of OC. However, the findings of the present study are mainly based on network pharmacology and bioinformatics analysis; therefore, further preclinical investigation is needed to verify the results.

## Data Availability

The datasets presented in this study can be found in online repositories. The names of the repository/repositories and accession number(s) can be found in the article/supplementary material.
